# Distribution of Major and trace elements in Koppunuru area, Guntur district, Andhra Pradesh, India

**DOI:** 10.1016/j.dib.2018.02.060

**Published:** 2018-03-01

**Authors:** K. Arumugam, S. Srinivasalu, R. Purvaja, R. Ramesh

**Affiliations:** a Institute for Ocean Management, Koodal Building, Anna University, Chennai 600025, India; b National Centre for Sustainable Coastal Management, Ministry of Environment, Forest and Climate Change (MoEF&CC), Anna University Campus, Chennai 600025, India

**Keywords:** Palnad sub-basin, Koppunuru, Geochemistry, Provenance, Uranium mineralization

## Abstract

From koppunuru study area totally 58 samples were collected in 7 different boreholes, minimum depth of 28 m and Maximum depth of 157.7 m. The borehole samples geochemical analysis (major and trace elements) was carried out at Atomic Minerals Directorate for Exploration & Research (AMD), Hyderabad, India. Major and trace element studies have been conducted on the Neoproterozoic Palnad sub-basin Andhra Pradesh, South India, to determine their Geochemistry, Uranium mineralization and provenance characteristics. Geochemically, this sedimentary basin has a different litho – unit like as gritty quartzite, conglomerate, and Shale. This study area mainly dominated by Uranium deposited and radioactive elements are predominately deposit. Strong positive correlation between Uranium and Lead (*r* = 0.887) suggested radiogenic nature of this system.

**Specifications Table**

**Table:** 

Subject area	Geochemistry
More specific subject area	Geochemistry, sedimentology,
Type of data	Table, graph, figure
How data was acquired	The sample pellets were analyzed using a The Magix Pro PW 2440 (PANalytical) WDXRF (Wavelength Dispersive X-Ray Fluorescence Spectroscopy). International rock standard GSR-4 and GSR-5 were used as reference material during major and trace element analyses.
Data format	Analyzed
Experimental factors	Samples were crushed and powered to 230 mesh size in an agate mill. Major and trace elements were determined from pressed pellets, which were prepared by using collapsible aluminum cups. These cups were filled with boric acid and about 1 g of each powdered rock sample was put on top of the boric acid and these cups were pressed under a hydraulic press at 20 t pressure.
Experimental features	Major and minor element studies of Koppunuru sediment samples.
Data source location	Koppunuru, Guntur District, Andhra Pradesh, South India.
Data accessibility	Data available within the article provide a direct URL to data

**Value of the data**

• Determine to the geochemical characteristics of Uranium bearing formations.
• Tectonic activity, weathering index of sediment and Provenance of Palnad sub basin.
• Characterization of unconformity uranium deposits in Kopunuru sediment.
• Relationship between major oxide and trace elements especially Uranium deposits.

## 1 Data

**Fig. 1:**
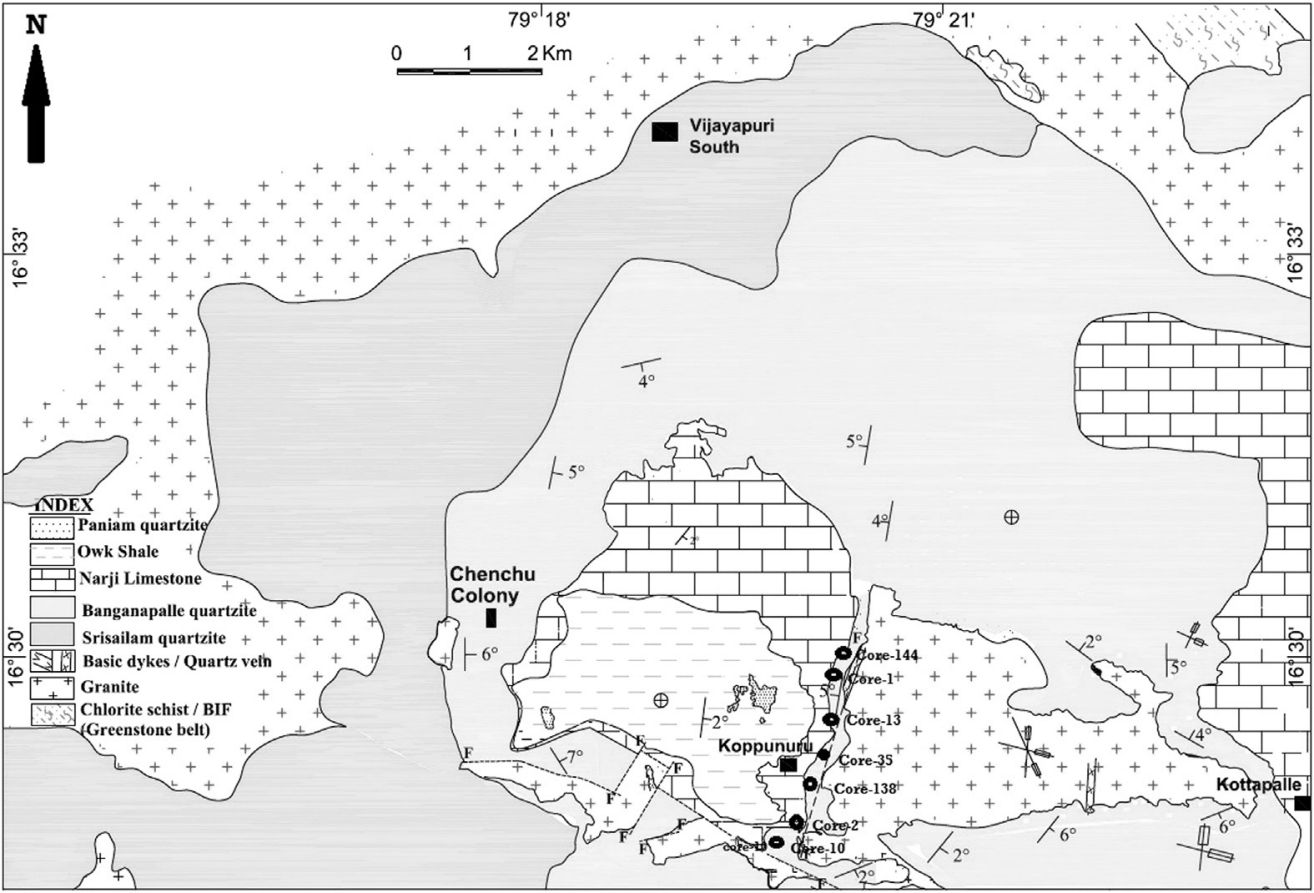
Geological and Study area map of Koppunuru Neo Proterozoic palnad-sub basin, Guntur District, Andhra Pradesh.

**Fig. 2:**
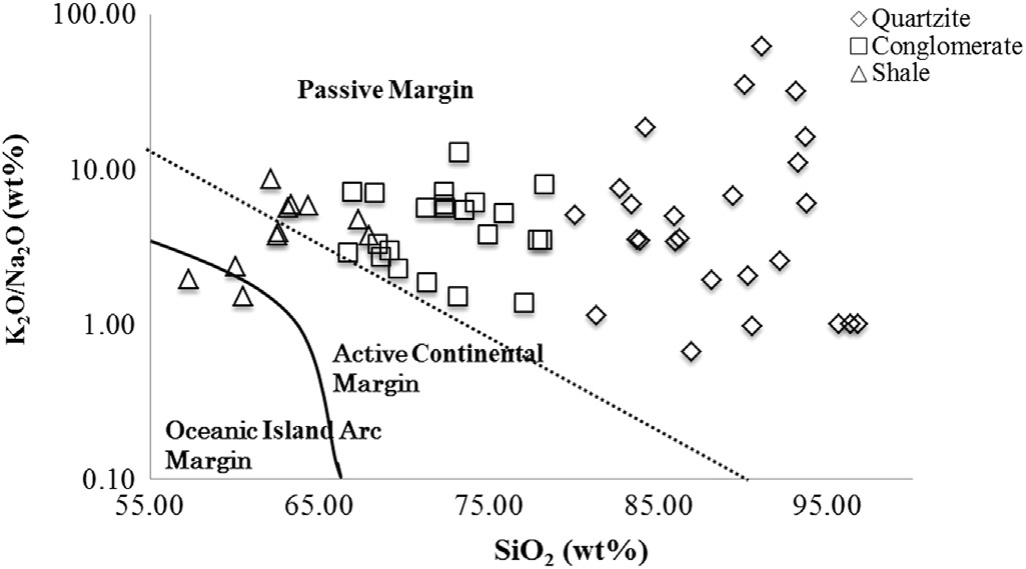
Discriminant diagram [1] showing deposition of Palnad sediments in a passive margin tectonic setting.

The sampling location was chosen from Koppunuru Neo Proterozoic palnad-sub basin, Guntur District, Andhra Pradesh (Fig. 1). Table 1, Table 2, Table 3 is representing the Concentration of Major oxide (wt. %) and Trace element (ppm) data from Gritty Quartzite, Conglomerate and Shale of Palnad Sub-basin, Andhra Pradesh (A.P). Table 4, Table 5, Table 6 is representing Pearson correlation coefficient for Gritty Quartzite, Conglomerate and Shale in Palnad sub-basin, A.P, South India. (*r* > 0.5 is significant at 95% confidence level). Discriminant diagram [1] showing deposition of Palnad sediments in a passive margin tectonic setting (Fig. 2).

**Table 1:** Concentration of Major oxide (wt%) and Trace element (ppm) data from Gritty Quartzite of Palnad Sub-basin, Andhra Pradesh.

**Types of rock**	**1**	**2**	**3**	**4**	**5**	**6**	**7**	**8**	**9**	**10**	**11**	**12**	**13**	**14**	**15**	**16**	**17**	**18**	**19**	**20**	**21**	**22**	**23**	**24**	**25**
**SiO_2_ (%)**	82.74	89.38	86.23	83.40	90.52	90.13	95.65	93.66	93.12	96.36	96.75	93.28	93.75	91.09	84.22	81.35	86.98	85.98	92.15	80.10	85.95	88.12	83.91	83.75	90.26
**TiO_2_**	0.19	0.09	0.26	0.42	0.10	0.09	0.04	0.09	0.09	0.02	0.02	0.10	0.10	0.15	0.27	0.03	0.03	0.05	0.02	0.04	0.02	0.03	0.04	0.09	0.02
**Al_2_O_3_**	8.54	4.93	6.75	8.40	1.82	1.57	0.01	0.79	2.20	0.01	0.01	1.59	1.25	3.71	7.21	0.58	0.49	6.31	3.78	11.68	7.04	5.90	8.65	10.17	5.91
**Fe_2_O_3_(t)**	1.52	2.05	1.38	2.05	1.86	1.92	1.33	1.67	2.53	1.16	0.95	2.37	2.62	2.68	3.53	0.73	0.88	0.91	0.87	2.32	0.99	3.34	1.86	1.47	1.49
**MgO**	0.49	0.64	0.54	0.76	0.78	0.86	0.27	0.69	0.65	0.31	0.26	0.56	0.36	0.88	1.04	0.94	0.51	0.52	0.26	0.22	0.35	0.13	0.04	0.12	0.01
**MnO**	0.12	0.14	0.09	0.13	0.11	0.16	0.16	0.16	0.13	0.17	0.18	0.14	0.16	0.11	0.12	0.09	0.12	0.10	0.13	0.11	0.12	0.13	0.12	0.11	0.14
**CaO**	0.26	0.22	0.46	0.26	2.21	3.45	0.62	2.11	0.01	0.01	0.01	0.01	0.01	0.01	0.13	9.54	5.62	1.46	0.05	0.34	0.01	0.01	0.17	0.01	0.01
**Na_2_O**	0.42	0.26	0.68	0.46	0.41	0.01	0.01	0.01	0.01	0.01	0.01	0.01	0.01	0.01	0.11	0.07	0.12	0.86	0.66	0.72	0.64	0.54	0.89	0.92	0.61
**K_2_O**	3.14	1.76	2.41	2.71	0.40	0.35	0.01	0.16	0.32	0.01	0.01	0.11	0.06	0.62	2.06	0.08	0.08	2.92	1.68	3.66	3.17	1.04	3.08	3.21	1.26
**P_2_O_5_**	0.01	0.01	0.04	0.07	0.04	0.03	0.01	0.04	0.09	0.01	0.01	0.01	0.02	0.01	0.04	0.04	0.05	0.07	0.01	0.36	0.02	0.05	0.25	0.07	0.06
**Total**	97.43	99.48	98.84	98.66	98.25	98.57	98.11	99.38	99.15	98.07	98.21	98.18	98.34	99.27	98.73	93.45	94.88	99.18	99.61	99.55	98.31	99.29	99.01	99.92	99.77
**SiO_2_/Al_2_O_3_ (CMI)**	9.69	18.13	12.77	9.93	49.74	57.41	9565.00	118.56	42.33	9636.00	9675.00	58.67	75.00	24.55	11.68	140.26	177.51	13.63	24.38	6.86	12.21	14.94	9.70	8.24	15.27
**K_2_O/Na_2_O**	7.48	6.77	3.54	5.89	0.98	35.00	1.00	16.00	32.00	1.00	1.00	11.00	6.00	62.00	18.73	1.14	0.67	3.40	2.55	5.08	4.95	1.93	3.46	3.49	2.07
**K_2_O/Al_2_O_3_**	0.37	0.36	0.36	0.32	0.22	0.22	1.00	0.20	0.15	1.00	1.00	0.07	0.05	0.17	0.29	0.14	0.16	0.46	0.44	0.31	0.45	0.18	0.36	0.32	0.21
**CIA**	69.09	68.76	65.53	71.01	37.60	29.18	1.54	25.73	86.61	25.00	25.00	92.44	93.98	85.29	75.81	5.65	7.77	54.63	61.26	71.22	64.83	78.77	67.63	71.07	75.87
**Al_2_O_3_/TiO_2_**	0.72	1.05	0.86	0.81	3.23	4.36	244.00	6.19	1.70	169.00	144.00	2.08	2.66	1.20	1.01	19.79	15.02	1.08	0.97	0.63	0.75	0.88	0.72	0.58	0.60
**Co (ppm)**	10	10	10	10	10	12	17	10	10	10	10	15	13	10	10	10	10	10	10	58	14	10	57	10	10
**Ni**	75	70	47	73	53	90	77	78	92	130	87	95	126	87	75	81	73	68	79	91	72	59	80	73	82
**Cu**	107	115	119	181	99	131	135	145	118	146	147	114	129	104	113	96	120	157	124	101	113	124	131	130	159
**Zn**	10	10	31	10	10	11	10	16	14	10	10	21	10	23	13	14	16	19	10	10	10	20	18	10	10
**Ga**	14	10	12	10	10	10	10	10	10	10	10	10	10	11	10	10	10	12	11	10	10	10	11	13	10
**Rb**	10	51	85	91	25	22	10	10	20	10	10	12	10	39	60	10	10	84	48	106	92	30	89	93	36
**Sr**	41	21	41	29	20	35	10	31	10	10	10	10	10	23	12	48	28	64	26	70	62	14	49	40	13
**Y**	10	10	12	10	10	10	19	10	10	17	40	40	14	10	10	10	10	17	10	169	81	10	23	10	10
**Zr**	118	143	77	66	32	29	24	40	44	23	28	53	46	50	135	28	24	64	26	166	90	37	59	74	27
**Nb**	10	10	10	10	10	10	10	10	10	15	20	35	22	11	60	10	10	10	10	80	39	10	15	10	10
**Ba**	385	276	275	245	112	116	112	149	128	89	50	135	110	147	195	138	194	398	282	620	535	168	427	399	156
**Ce**	10	10	10	16	16	10	10	10	10	10	10	10	10	10	15	10	10	10	10	25	10	14	10	10	10
**Pb**	53	16	57	79	56	73	148	119	57	120	359	511	236	76	18	26	77	98	44	1297	660	39	445	45	31
**Th**	10	10	10	15	316	10	10	10	10	10	10	10	10	10	53	10	10	10	10	10	10	10	10	10	10
**U**	195	49	262	509	179	277	751	179	123	746	2063	2548	796	157	30	81	292	419	124	9458	4697	24	1639	107	49
**U/Th**	20	5	26	34	1	28	75	18	12	75	206	255	80	16	1	8	29	42	12	946	470	2	164	11	5

**Table 2:** Concentration of Major oxide (wt%) and Trace element (ppm) data from Conglomerate of Palnad Sub-basin, Andhra Pradesh.

**Types of rock**	**1**	**2**	**3**	**4**	**5**	**6**	**7**	**8**	**9**	**10**	**11**	**12**	**13**	**14**	**15**	**16**	**17**	**18**	**19**	**20**	**21**	**22**
**SiO_2_ (%)**	78.26	72.36	71.25	75.85	74.93	74.16	69.15	66.67	69.66	71.35	68.62	73.54	72.38	77.09	73.19	78.15	68.45	77.91	73.20	72.36	68.24	66.95
**TiO_2_**	0.36	0.51	0.66	0.47	0.25	0.37	0.37	0.18	0.16	0.12	0.12	0.47	0.46	0.10	0.12	0.11	0.18	0.13	0.10	0.13	0.14	0.20
**Al_2_O_3_**	10.70	13.77	15.62	12.07	12.18	14.60	17.27	19.42	17.02	16.14	17.42	13.84	13.09	11.67	14.92	11.27	19.31	12.85	17.52	18.04	20.50	20.50
**Fe_2_O_3_(t)**	5.19	8.09	6.37	6.38	3.98	4.87	4.01	1.76	1.75	1.69	1.88	5.19	5.64	1.73	2.37	1.74	2.65	2.15	2.05	1.72	2.13	3.22
**MgO**	1.24	1.03	0.96	0.76	0.62	0.67	1.00	0.59	0.43	0.36	0.49	0.83	0.80	0.57	0.50	0.59	0.73	0.35	0.48	0.60	0.61	0.81
**MnO**	0.14	0.12	0.10	0.12	0.13	0.10	0.12	0.10	0.11	0.10	0.10	0.10	0.11	0.10	0.11	0.08	0.12	0.11	0.11	0.09	0.12	0.11
**CaO**	0.09	0.01	0.02	0.14	3.22	0.16	1.19	1.23	1.22	1.12	1.42	1.53	2.47	0.29	0.33	0.41	0.03	0.12	0.01	0.01	0.01	0.01
**Na_2_O**	0.34	0.54	0.74	0.60	0.92	0.65	1.59	2.34	2.70	3.08	2.40	0.61	0.54	3.18	3.13	1.40	1.89	1.35	0.42	0.80	0.97	0.95
**K_2_O**	2.72	3.22	4.18	3.13	3.49	3.97	4.76	6.79	6.21	5.77	6.51	3.35	3.06	4.39	4.75	4.90	6.28	4.76	5.39	5.74	6.81	6.83
**P_2_O_5_**	0.02	0.08	0.06	0.07	0.19	0.06	0.08	0.26	0.17	0.15	0.49	0.06	0.06	0.05	0.10	0.03	0.08	0.07	0.06	0.10	0.08	0.10
**Total**	99.06	99.73	99.96	99.59	99.91	99.61	99.54	99.34	99.43	99.88	99.45	99.52	98.61	99.17	99.52	98.68	99.72	99.80	99.34	99.59	99.61	99.68
**SiO_2_/Al_2_O_3_ (CMI)**	7.31	5.25	4.56	6.28	6.15	5.08	4.00	3.43	4.09	4.42	3.94	5.31	5.53	6.61	4.91	6.93	3.54	6.06	4.18	4.01	3.33	3.27
**K_2_O/Na_2_O**	8.00	5.96	5.65	5.22	3.79	6.11	2.99	2.90	2.30	1.87	2.71	5.49	5.67	1.38	1.52	3.50	3.32	3.53	12.83	7.18	7.02	7.19
**K_2_O/Al_2_O_3_**	0.25	0.23	0.27	0.26	0.29	0.27	0.28	0.35	0.36	0.36	0.37	0.24	0.23	0.38	0.32	0.43	0.33	0.37	0.31	0.32	0.33	0.33
**CIA**	77.26	78.51	75.97	75.72	61.48	75.34	69.61	65.21	62.69	61.82	62.77	71.60	68.32	59.75	64.50	62.68	70.19	67.35	75.06	73.36	72.46	72.46
**Al_2_O_3_/TiO_2_**	0.94	0.98	0.83	0.96	1.04	0.74	0.76	0.67	0.74	0.76	0.74	0.87	1.00	0.89	0.76	0.82	0.62	0.70	0.49	0.50	0.53	0.59
**Co (ppm)**	10	15	15	10	10	95	10	10	10	10	10	10	10	10	10	10	22	10	23	10	10	10
**Ni**	68	110	107	103	68	189	64	55	60	51	68	70	63	56	67	49	74	72	72	91	54	65
**Cu**	123	88	88	95	91	183	129	93	99	92	105	88	87	120	110	87	97	101	108	85	103	96
**Zn**	10	40	42	25	35	10	55	10	10	10	10	51	37	16	20	16	10	13	26	15	11	14
**Ga**	10	15	16	14	14	14	18	17	17	15	16	17	15	15	16	15	16	14	15	15	17	16
**Rb**	138	93	124	92	99	114	129	153	142	149	188	94	88	127	137	165	181	77	155	166	197	198
**Sr**	17	21	38	27	63	60	79	82	91	86	93	42	31	128	129	85	96	69	50	61	78	82
**Y**	11	10	10	10	10	85	10	27	24	19	30	10	10	10	13	11	19	11	34	92	10	24
**Zr**	80	120	228	142	103	282	131	129	158	102	117	152	114	70	85	87	147	79	127	101	64	181
**Nb**	43	18	14	16	10	52	10	10	10	10	10	11	10	10	10	10	11	10	14	22	10	10
**Ba**	182	249	376	279	369	587	416	554	557	507	517	551	297	489	491	436	670	451	517	440	605	567
**Ce**	19	24	42	19	10	34	16	63	51	28	47	19	30	10	24	63	74	16	24	67	10	46
**Pb**	13	19	21	17	13	687	26	50	60	46	43	11	13	57	48	33	129	56	228	408	75	80
**Th**	10	10	11	10	10	10	17	21	26	22	23	10	10	10	19	19	47	20	10	15	16	54
**U**	10	17	17	25	20	4038	43	58	48	37	35	36	28	144	68	20	298	88	1165	2409	90	82
**U/Th**	1	2	2	3	2	404	3	3	2	2	2	4	3	14	4	1	6	4	117	161	6	2

**Table 3:** Concentration of Major oxide (wt%) and Trace element (ppm) data from Shale of Palnad Sub-basin, Andhra Pradesh.

**Types of rock**	**1**	**2**	**3**	**4**	**5**	**6**	**7**	**8**	**9**	**10**	**11**
**SiO_2_ (%)**	63.15	67.29	63.34	62.55	57.26	60.06	60.51	64.35	62.54	67.96	62.13
**TiO_2_**	0.77	0.71	0.82	0.24	0.07	0.18	0.36	0.71	0.78	0.73	0.74
**Al_2_O_3_**	19.74	17.82	20.47	21.53	26.01	22.09	17.97	19.66	17.44	16.99	20.36
**Fe_2_O_3_(t)**	6.61	6.54	7.49	3.02	1.53	2.59	4.17	7.95	10.28	7.17	6.59
**MgO**	1.35	1.10	1.35	0.81	1.00	0.96	1.43	1.33	1.12	1.22	1.47
**MnO**	0.11	0.09	0.09	0.08	0.08	0.08	0.09	0.06	0.10	0.08	0.08
**CaO**	1.59	0.37	0.06	0.13	0.48	0.57	2.10	0.06	1.41	0.22	2.06
**Na_2_O**	0.84	0.93	0.84	2.12	4.26	2.92	3.11	0.77	1.11	1.05	0.56
**K_2_O**	4.77	4.45	4.97	8.28	8.30	6.89	4.74	4.47	4.13	3.96	4.85
**P_2_O_5_**	0.11	0.08	0.07	0.04	0.10	0.11	0.12	0.07	0.09	0.07	0.09
**Total**	99.04	99.38	99.50	98.80	99.09	96.45	94.60	99.43	99.00	99.45	98.93
**SiO_2_/Al_2_O_3_ (CMI)**	3.20	3.78	3.09	2.91	2.20	2.72	3.37	3.27	3.59	4.00	3.05
**K_2_O/Na_2_O**	5.68	4.78	5.92	3.91	1.95	2.36	1.52	5.81	3.72	3.77	8.66
**K_2_O/Al_2_O_3_**	0.24	0.25	0.24	0.38	0.32	0.31	0.26	0.23	0.24	0.23	0.24
**CIA**	73.27	75.60	77.71	67.16	66.61	68.03	64.36	78.77	72.40	76.46	73.16
**Al_2_O_3_/TiO_2_**	0.81	0.80	0.76	0.68	0.60	0.64	0.89	0.78	1.09	0.85	0.80
**Co (ppm)**	10	12	20	10	10	10	12	10	12	10	13
**Ni**	86	78	94	50	46	49	48	63	66	66	77
**Cu**	77	159	108	110	95	88	92	66	58	65	89
**Zn**	60	220	73	21	36	44	70	61	50	58	69
**Ga**	19	25	19	12	17	17	18	17	16	18	19
**Rb**	155	147	154	71	290	241	51	132	103	113	134
**Sr**	58	50	41	125	218	165	120	21	32	34	71
**Y**	15	13	14	12	10	31	21	10	10	12	17
**Zr**	173	221	255	58	52	139	123	159	179	210	149
**Nb**	16	14	15	10	10	10	10	11	16	10	11
**Ba**	449	381	441	617	790	765	538	295	292	321	335
**Ce**	33	32	25	83	11	41	48	29	29	17	51
**Pb**	19	30	27	77	57	127	47	15	19	13	23
**Th**	17	10	13	10	10	21	22	13	14	10	15
**U**	14	15	15	71	55	539	127	17	18	16	31
**U/Th**	1	2	1	7	6	26	6	1	1	2	2

**Table 4:** Pearson correlation coefficient for Gritty quartzite in Palnad sub-basin,AP, South India.

	SiO_2_	TiO_2_	Al_2_O_3_	Fe_2_O_3_(t)	MgO	MnO	CaO	Na_2_O	K_2_O	P_2_O_5_	Co	Ni	Cu	Zn	Ga	Rb	Sr	Y	Zr	Nb	Ba	Ce	Pb	Th	U
SiO_2_	1																								
TiO_2_	− 0.31	1																							
Al_2_O_3_	− 0.77	0.34	1																						
Fe_2_O_3_(t)	− 0.03	0.37	0.22	1																					
MgO	− 0.10	0.51	− 0.26	0.23	1																				
MnO	0.77	− 0.22	− 0.54	0.03	− 0.21	1																			
CaO	− 0.27	− 0.19	− 0.39	− 0.39	0.43	− 0.29	1																		
Na_2_O	− 0.59	− 0.01	0.80	− 0.19	− 0.52	− 0.56	− 0.25	1																	
K_2_O	− 0.75	0.28	0.95	− 0.01	− 0.26	− 0.54	− 0.34	0.84	1																
P_2_O_5_	− 0.52	− 0.10	0.57	0.16	− 0.32	− 0.30	− 0.06	0.48	0.51	1															
Co	− 0.37	− 0.20	0.45	0.12	− 0.39	− 0.14	− 0.13	0.38	0.43	0.91	1														
Ni	0.43	− 0.24	− 0.36	0.08	− 0.12	0.58	− 0.11	− 0.48	− 0.38	0.01	0.11	1													
Cu	0.25	0.18	− 0.01	− 0.20	− 0.28	0.45	− 0.26	0.11	0.02	− 0.09	− 0.16	0.14	1												
Zn	− 0.08	0.19	0.03	0.16	0.16	− 0.43	0.03	0.09	− 0.01	0.00	− 0.01	− 0.34	− 0.15	1											
Ga	− 0.38	0.20	0.49	− 0.22	− 0.16	− 0.43	− 0.17	0.50	0.56	− 0.03	− 0.05	− 0.28	− 0.07	0.19	1										
Rb	− 0.64	0.28	0.84	0.03	− 0.20	− 0.54	− 0.31	0.80	0.87	0.56	0.46	− 0.37	0.10	0.12	0.27	1									
Sr	− 0.73	− 0.06	0.58	− 0.35	− 0.06	− 0.60	0.24	0.63	0.71	0.54	0.47	− 0.30	− 0.16	0.10	0.38	0.66	1								
Y	− 0.29	− 0.23	0.39	0.02	− 0.28	− 0.09	− 0.16	0.25	0.39	0.68	0.67	0.13	− 0.25	− 0.16	− 0.17	0.43	0.51	1							
Zr	− 0.59	0.34	0.71	0.34	0.10	− 0.34	− 0.30	0.32	0.70	0.42	0.37	− 0.17	− 0.32	− 0.07	0.25	0.56	0.43	0.51	1						
Nb	− 0.31	0.03	0.38	0.36	0.00	− 0.09	− 0.21	0.07	0.34	0.53	0.53	0.19	− 0.32	− 0.14	− 0.24	0.37	0.26	0.81	0.65	1					
Ba	− 0.71	− 0.01	0.83	− 0.13	− 0.32	− 0.52	− 0.20	0.77	0.91	0.62	0.58	− 0.26	− 0.16	− 0.04	0.43	0.80	0.84	0.62	0.68	0.45	1				
Ce	− 0.44	0.22	0.47	0.40	0.03	− 0.23	− 0.10	0.24	0.36	0.67	0.51	− 0.11	− 0.17	− 0.18	− 0.23	0.41	0.27	0.67	0.51	**0.69**	0.38	1			
Pb	− 0.22	− 0.24	0.33	0.04	− 0.33	− 0.02	− 0.20	0.22	0.34	0.70	0.76	0.22	− 0.21	− 0.10	− 0.19	0.39	0.47	0.96	0.43	**0.77**	**0.58**	**0.58**	1		
Th	0.04	0.07	− 0.12	0.09	0.27	− 0.18	0.09	0.03	− 0.14	− 0.05	− 0.09	− 0.32	− 0.28	− 0.15	− 0.12	− 0.09	− 0.13	− 0.09	− 0.09	− 0.03	− 0.18	0.31	− 0.12	1	
U	− 0.27	− 0.22	0.37	0.02	− 0.29	− 0.06	− 0.17	0.23	0.38	0.68	0.70	0.17	− 0.23	− 0.16	− 0.19	0.42	0.50	0.99	0.49	**0.80**	**0.61**	**0.64**	**0.98**	− 0.10	1

(*r* > 0.5 is significant at 95 % confidence level; *n* = 25)

**Table 5:** Pearson correlation coefficient for Conglomerate in Palnad sub-basin, AP, South India.

	SiO_2_	TiO_2_	Al_2_O_3_	Fe_2_O_3_(t)	MgO	MnO	CaO	Na_2_O	K_2_O	P_2_O_5_	Co	Ni	Cu	Zn	Ga	Rb	Sr	Y	Zr	Nb	Ba	Ce	Pb	Th	U
SiO_2_	1																								
TiO_2_	0.07	1																							
Al_2_O_3_	− 0.91	− 0.30	1																						
Fe_2_O_3_(t)	0.17	**0.94**	− 0.40	1																					
MgO	0.04	**0.75**	− 0.20	**0.76**	1																				
MnO	0.02	0.24	− 0.10	0.39	0.47	1																			
CaO	− 0.09	0.07	− 0.18	0.02	− 0.11	0.10	1																		
Na_2_O	− 0.21	− 0.58	0.16	− 0.64	− 0.57	− 0.28	0.10	1																	
K_2_O	− 0.70	− 0.68	**0.85**	− 0.76	− 0.53	− 0.31	− 0.18	0.48	1																
P_2_O_5_	− 0.48	− 0.30	0.34	− 0.33	− 0.37	− 0.13	0.41	0.42	0.47	1															
Co	0.07	0.15	− 0.01	0.17	0.00	− 0.12	− 0.20	− 0.22	− 0.12	− 0.15	1														
Ni	0.14	0.49	− 0.12	**0.52**	0.23	− 0.03	− 0.29	− 0.43	− 0.35	− 0.18	**0.84**	1													
Cu	0.14	0.00	− 0.08	0.03	0.08	0.11	− 0.21	0.00	− 0.12	− 0.12	**0.80**	**0.59**	1												
Zn	0.04	**0.68**	− 0.23	**0.62**	0.50	0.14	0.34	− 0.39	− 0.55	− 0.25	− 0.17	0.06	− 0.18	1											
Ga	− 0.72	− 0.09	**0.64**	− 0.26	− 0.25	− 0.35	0.15	0.36	**0.56**	0.27	− 0.17	− 0.24	− 0.21	0.27	1										
Rb	− 0.56	− 0.57	**0.71**	− 0.60	− 0.18	− 0.19	− 0.30	0.27	**0.82**	0.32	− 0.10	− 0.32	− 0.05	− 0.54	0.28	1									
Sr	− 0.24	− 0.74	0.31	− 0.77	− 0.61	− 0.33	− 0.02	**0.86**	**0.62**	0.30	− 0.09	− 0.38	0.12	− 0.42	0.44	0.47	1								
Y	− 0.10	− 0.19	0.29	− 0.21	− 0.21	− 0.39	− 0.22	− 0.13	0.23	0.10	**0.61**	**0.57**	0.41	− 0.34	− 0.08	0.22	0.00	1							
Zr	− 0.27	**0.51**	0.20	0.39	0.22	− 0.16	− 0.10	− 0.27	− 0.05	− 0.04	**0.70**	**0.74**	0.41	0.15	0.15	− 0.09	− 0.23	0.41	1						
Nb	0.33	0.28	− 0.26	0.34	0.39	0.17	− 0.31	− 0.42	− 0.39	− 0.26	**0.73**	**0.73**	**0.71**	− 0.21	− 0.63	− 0.13	− 0.41	**0.55**	0.42	1					
Ba	− 0.52	− 0.54	**0.67**	− 0.64	− 0.57	− 0.38	− 0.11	0.43	**0.76**	0.24	0.27	− 0.07	0.17	− 0.39	**0.60**	**0.55**	**0.66**	0.29	0.23	− 0.23	1				
Ce	− 0.40	− 0.21	0.44	− 0.32	− 0.13	− 0.49	− 0.15	0.15	**0.53**	0.26	0.05	0.00	− 0.24	− 0.38	0.23	**0.52**	0.19	0.42	0.26	− 0.04	0.36	1			
Pb	0.02	− 0.11	0.19	− 0.10	− 0.16	− 0.29	− 0.31	− 0.21	0.10	− 0.12	**0.85**	**0.73**	**0.64**	− 0.30	− 0.13	0.11	− 0.01	**0.92**	**0.52**	**0.67**	0.34	0.24	1		
Th	− 0.56	− 0.38	**0.60**	− 0.38	− 0.15	− 0.02	− 0.18	0.29	**0.68**	0.19	− 0.12	− 0.26	− 0.16	− 0.40	0.31	**0.61**	0.44	0.00	0.12	− 0.30	**0.55**	**0.54**	− 0.07	1	
U	0.09	− 0.04	0.10	− 0.03	− 0.11	− 0.29	− 0.26	− 0.26	− 0.01	− 0.14	**0.84**	**0.76**	**0.64**	− 0.22	− 0.18	0.02	− 0.08	**0.91**	**0.52**	**0.70**	0.24	0.19	**0.99**	− 0.17	1

(*r* > 0.5 is significant at 95 % confidence level; *n* = 22)

**Table 6:** Pearson correlation coefficient for Shale in Palnad sub-basin,AP, South India.

	**SiO_2_**	**TiO_2_**	**Al_2_O_3_**	**Fe_2_O_3_(t)**	**MgO**	**MnO**	**CaO**	**Na_2_O**	**K_2_O**	**P_2_O_5_**	**Co**	**Ni**	**Cu**	**Zn**	**Ga**	**Rb**	**Sr**	**Y**	**Zr**	**Nb**	**Ba**	**Ce**	**Pb**	**Th**	**U**
**SiO_2_**	1																								
**TiO_2_**	**0.71**	1																							
**Al_2_O_3_**	− 0.75	− 0.70	1																						
**Fe_2_O_3_(t)**	**0.62**	**0.93**	− 0.73	1																					
**MgO**	0.18	**0.63**	− 0.42	0.50	1																				
**MnO**	0.00	0.28	− 0.27	0.23	0.12	1																			
**CaO**	− 0.31	0.12	− 0.23	0.11	**0.53**	0.49	1																		
**Na_2_O**	− 0.77	− 0.93	**0.64**	− 0.84	− 0.47	− 0.13	0.00	1																	
**K_2_O**	− 0.66	− 0.87	**0.86**	− 0.85	− 0.72	− 0.23	− 0.29	**0.73**	1																
**P_2_O_5_**	− 0.49	− 0.18	0.06	− 0.18	0.35	0.40	**0.68**	0.38	− 0.14	1															
**Co**	0.07	0.43	− 0.10	0.33	0.40	0.20	− 0.05	− 0.32	− 0.26	− 0.13	1														
**Ni**	**0.53**	**0.86**	− 0.37	**0.66**	**0.53**	0.42	0.03	− 0.81	− 0.59	− 0.13	**0.62**	1													
**Cu**	0.15	− 0.14	0.11	− 0.31	− 0.26	0.07	− 0.24	0.07	0.23	− 0.18	0.27	0.15	1												
**Zn**	**0.55**	0.38	− 0.42	0.25	0.14	0.16	− 0.06	− 0.34	− 0.43	0.02	0.19	0.42	**0.72**	1											
**Ga**	0.43	0.45	− 0.33	0.26	0.41	0.24	0.10	− 0.33	− 0.54	0.30	0.27	**0.55**	**0.52**	**0.90**	1										
**Rb**	− 0.46	− 0.41	**0.75**	− 0.44	− 0.30	− 0.12	− 0.27	0.44	0.47	0.32	− 0.10	− 0.11	0.08	− 0.06	0.15	1									
**Sr**	− 0.81	− 0.95	**0.80**	− 0.93	− 0.51	− 0.13	0.02	**0.93**	**0.86**	0.33	− 0.31	− 0.71	0.17	− 0.34	− 0.31	**0.56**	1								
**Y**	− 0.32	− 0.36	0.09	− 0.42	0.00	0.04	0.28	0.31	0.13	**0.55**	− 0.03	− 0.24	0.05	− 0.07	0.05	0.19	0.38	1							
**Zr**	**0.70**	**0.83**	− 0.65	**0.74**	0.48	0.27	− 0.11	− 0.73	− 0.80	− 0.06	**0.58**	**0.80**	0.08	**0.53**	**0.63**	− 0.18	− 0.78	− 0.05	1						
**Nb**	0.27	**0.68**	− 0.35	**0.67**	0.24	**0.73**	0.14	− 0.56	− 0.48	0.07	0.43	**0.76**	0.05	0.33	0.37	− 0.09	− 0.56	− 0.28	**0.61**	1					
**Ba**	− 0.74	− 0.92	**0.79**	− 0.93	− 0.58	− 0.06	− 0.14	**0.88**	**0.87**	0.27	− 0.22	− 0.61	0.22	− 0.34	− 0.32	**0.58**	**0.95**	0.46	− 0.67	− 0.45	1				
**Ce**	− 0.09	− 0.26	− 0.01	− 0.28	− 0.24	− 0.05	0.19	0.00	0.34	− 0.31	− 0.12	− 0.24	0.23	− 0.17	− 0.44	− 0.52	0.13	0.25	− 0.42	− 0.26	0.15	1			
**Pb**	− 0.55	− 0.82	**0.54**	− 0.77	− 0.65	− 0.18	− 0.16	**0.67**	**0.73**	0.16	− 0.25	− 0.62	0.20	− 0.27	− 0.35	0.39	**0.76**	0.71	− 0.52	− 0.48	**0.85**	0.39	1		
**Th**	− 0.41	− 0.16	− 0.10	− 0.14	0.36	0.24	**0.63**	0.22	− 0.14	**0.75**	− 0.01	− 0.17	− 0.25	− 0.19	− 0.02	− 0.07	0.18	**0.81**	− 0.04	− 0.05	0.22	0.19	0.37	1	
**U**	− 0.41	− 0.58	0.29	− 0.53	− 0.37	− 0.15	− 0.02	0.48	0.38	0.39	− 0.22	− 0.48	− 0.02	− 0.21	− 0.17	0.39	**0.54**	**0.89**	− 0.23	− 0.39	**0.63**	0.18	**0.89**	**0.61**	1

(*r* > 0.5 is significant at 95 % confidence level; *n* = 11)

## 2 Experimental design, materials and methods

Sediment samples were crushed and powered to 230 mesh size in an agate mill. Major and trace elements were determined from pressed pellets, which were prepared by using collapsible aluminum cups. These cups were filled with boric acid and about 1 g of each powdered rock sample was put on top of the boric acid and these cups were pressed under a hydraulic press at 20 t pressure. The sample pellets were analyzed using a The Magix Pro PW 2440 (PANalytical) WDXRF (Wavelength Dispersive X-Ray Fluorescence Spectroscopy). International rock standard GSR-4 and GSR-5 were used as reference material during major and trace element analyses. The precision and accuracy of the data were well within the standards and an analytical precision (< 5%. % of RSD) was observed for major oxides whereas % error in accuracy of analysis was 1%. The % of RSD for trace elements was within 1–5%, whereas accuracy (% error) was within 5–10%.

The Palnad sub - basin has shown immense potential of uranium mineralization in overall samples (10–9458 ppm. Average – 611 ppm). Uranium mineralization occurs as stringers, veins and along cavities and as grain boundary fillings. Pitchblende, coffinite and mixed phases of U, Ti, Si, Al, Ca, P and Pb constitute radioactive phases of ore body. Pitchblende and coffinite contain almost nil amounts of Thorium [2]. SiO_2_ vs. K_2_O/Na_2_O discriminant diagram showing deposition of Palnad sediments are in passive margin tectonic setting, the shale only is showing Active Continental Margin and Oceanic Island Arc Margin (Fig. 2) [1].
